# A decision support system for electrode shaping in multi-pad FES foot drop correction

**DOI:** 10.1186/s12984-017-0275-5

**Published:** 2017-07-03

**Authors:** Jovana Malešević, Suzana Dedijer Dujović, Andrej M. Savić, Ljubica Konstantinović, Aleksandra Vidaković, Goran Bijelić, Nebojša Malešević, Thierry Keller

**Affiliations:** 1 Tecnalia Serbia Ltd., Belgrade, Serbia; 2 University of Belgrade, Belgrade, Serbia; 3 Clinic for Rehabilitation “Dr Miroslav Zotović”, Belgrade, Serbia; 4 University of Belgrade, School of Electrical Engineering, Belgrade, Serbia; 5 University of Belgrade, Faculty of Medicine, Belgrade, Serbia; 6 Tecnalia Research & Innovation - Health Division, Donostia-San Sebastián, Spain; 7 Department of Biomedical Engineering, Lund University, Lund, Sweden

**Keywords:** Decision support system, Foot drop, Functional electrical stimulation, Multi-pad electrode, Stroke

## Abstract

**Background:**

Functional electrical stimulation (FES) can be applied as an assistive and therapeutic aid in the rehabilitation of foot drop. Transcutaneous multi-pad electrodes can increase the selectivity of stimulation; however, shaping the stimulation electrode becomes increasingly complex with an increasing number of possible stimulation sites. We described and tested a novel decision support system (DSS) to facilitate the process of multi-pad stimulation electrode shaping. The DSS is part of a system for drop foot treatment that comprises a custom-designed multi-pad electrode, an electrical stimulator, and an inertial measurement unit.

**Methods:**

The system was tested in ten stroke survivors (3–96 months post stroke) with foot drop over 20 daily sessions. The DSS output suggested stimulation pads and parameters based on muscle twitch responses to short stimulus trains. The DSS ranked combinations of pads and current amplitudes based on a novel measurement of the quality of the induced movement and classified them based on the movement direction (dorsiflexion, plantar flexion, eversion and inversion) of the paretic foot. The efficacy of the DSS in providing satisfactory pad-current amplitude choices for shaping the stimulation electrode was evaluated by trained clinicians. The range of paretic foot motion was used as a quality indicator for the chosen patterns.

**Results:**

The results suggest that the DSS output was highly effective in creating optimized FES patterns. The position and number of pads included showed pronounced inter-patient and inter-session variability; however, zones for inducing dorsiflexion and plantar flexion within the multi-pad electrode were clearly separated. The range of motion achieved with FES was significantly greater than the corresponding active range of motion (*p* < 0.05) during the first three weeks of therapy.

**Conclusions:**

The proposed DSS in combination with a custom multi-pad electrode design covering the branches of peroneal and tibial nerves proved to be an effective tool for producing both the dorsiflexion and plantar flexion of a paretic foot. The results support the use of multi-pad electrode technology in combination with automatic electrode shaping algorithms for the rehabilitation of foot drop.

**Trial registration:**

This study was registered at the Current Controlled Trials website with ClinicalTrials.gov ID NCT02729636 on March 29, 2016.

## Background

Foot drop is the inability or difficulty to voluntarily lift the foot due to weak or absent ankle dorsiflexors. It is commonly caused by stroke, multiple sclerosis and spinal cord trauma [1]. To avoid foot dragging during the swing phase of the gait, patients with foot drop adopt abnormal gait patterns characterized by hip hitching, circumduction and toe catch. These distortions of the gait pattern lead to a decrease in the gait velocity and walking endurance, longer stance and double support gait phases, an increased energy cost, instability and a tendency to trip and fall [2].

Functional electrical stimulation (FES) is an active approach for treating foot drop. It is a technique that relies on the production of short bursts of electrical pulses to induce contraction by eliciting an action potential in the motor neurons that innervate a muscle [3]. FES principles can be employed for the therapeutic treatment of foot drop and/or in the form of an active assistive orthotic device for daily use and the long-term replacement of the impaired motor function [2, 4–7]. FES-based therapeutic and assistive devices for foot drop correction typically stimulate the common peroneal nerve in the swing phase of the gait to ensure foot lifting, i.e., the dorsiflexion (DF) of the ankle (for a review, see references [8, 9]). Moreover, producing plantar flexion (PF) with FES during the pre-swing phase of the gait results in better knee flexion, which also facilitates the swing phase [10].

The most common challenges in foot drop FES applications are determining motor points, i.e., adequately positioning the electrodes, setting stimulation parameters that produce comfortable foot lift without unnatural foot eversion or inversion, and increased muscle fatigue from electrically induced contraction [11–13]. Currently available commercial systems stimulate dorsiflexion only; they do not compensate for eversion (EV) or inversion (IV) and do not support push-off due to the complexity of proper electrode positioning [11, 14–16].

One of the options for overcoming these issues is the use of multi-pad surface electrodes. Multi-pad electrodes comprise many relatively small stimulation pads. Each pad can be activated separately or as a part of a stimulation pattern, i.e., a set of individual pads that are associated with different parameters, including the pulse width and amplitude [17–20]. Multi-pad systems aim to improve the selectivity of stimulation and provide easier and faster electrode donning and doffing [21, 22].

However, many pads increase the number of possible combinations for stimulation electrode shaping. Defining the stimulation patterns can be time consuming and laborious and requires medical training and knowledge of neurophysiology and anatomy. Therefore, novel FES systems may benefit greatly from an automated stimulation electrode shaping process that does not rely on precise or finely reproducible electrode positioning. Such an automated process can reduce the clinicians’ time and effort in therapeutic applications of FES and increase the users’ independence in terms of the everyday use of a FES system in assistive applications.

Several groups have investigated the design of multi-pad stimulation systems and control algorithms for the automatic shaping of stimulation patterns/electrodes [14, 23–25]. Elsaify presented a proof of concept for using muscle twitches for the selection of stimulation patterns for DF induction using multiple individual electrodes positioned over the tibialis anterior (TA) muscle and an inertial sensor on the foot for recording the twitch responses [24]. Heller et al. described the principle of searching for an optimal 4 × 4 pad electrode within an 8 × 8 pad cathode placed over the peroneal nerve, with a common anode over the TA muscle [25]. More recently, Valtin et al. described a FES system for foot drop with the control of DF and EV during the swing phase of the gait by two decoupled iterative learning controllers. They employed two automatically tuned multi-pad electrodes, one over the TA muscle and the other over the area of the peroneal nerve. Foot movements were monitored with 2 inertial measurement units at the shank and foot [14]. In a recent publication, Kenney et al. described a ShefStim device [15, 26] array-based FES system for the correction of foot drop that comprises a three-phase search algorithm for finding an appropriate candidate out of 25 stimulation patterns within a multi-pad array. Prenton et al. tested the automatic algorithm for stimulation pattern selection described by Kenney et al. [15] for unsupervised use by individuals with foot drop [27].

Although FES-induced ankle DF can correct foot drop, it also decreases knee flexion and ankle plantar flexion at the toe-off in the swing phase of the gait, which decreases the propulsive force generated during the transition from the stance phase to the swing phase [28], which in turn implies a need for more complex stimulation patterns in systems for FES-assisted gait, including the support of movements other than DF. To the best of our knowledge, none of the currently available multi-pad FES systems for foot drop treatment support the induction of both DF and PF movement. An assistive benefit of introducing electrically induced PF during FES-assisted walking is the enhancement of the propulsive force during the push-off phase. In addition to assistive effects, the therapeutic effects of FES that presumably arise through the facilitation of neural plasticity by increasing the strength of afferent inputs are also important to consider when designing a closed-loop FES system. In particular, an FES system in which the timing of the electrical input that creates the afferent feedback is synchronized with the electrophysiological correlate of voluntary movement (i.e., EMG or a position sensor) has been shown to facilitate neural plasticity (for a review, see [29]). Therefore, creating a natural, temporally precise sequence of phases (i.e., a FES-induced DF in the swing phase and a PF in the push-off phase of the gait) during FES-assisted walking therapy may further enhance motor recovery via the synchronization of sensory and motor information.

Our goal was to clinically test a novel foot drop device supporting the induction of both DF and PF movements. Because the system was based on multi-pad technology, we introduced and described a dedicated decision support system (DSS) to facilitate the process of defining the stimulation patterns for inducing ankle DF and PF movements. The novel foot drop device that was the focus of this study comprised a custom-designed multi-pad electrode, an electrical stimulator, and a single inertial sensor (Fesia Walk, Tecnalia R&I, Donostia/San Sebastián, Spain). The novelty of the applied methodology lies in the multi-pad electrode design and positioning, covering the branches of peroneal and tibial nerves for supporting both DF and PF and a dedicated DSS for easier shaping of the DF and PF stimulation patterns.

An additional goal was the clinical evaluation of the performance of the DSS for the automatic identification of high-quality pads for further electrode shaping. A qualitative assessment of the DSS output was conducted by comparing the pads suggested by the DSS with the pads selected by trained rehabilitation specialists. Moreover, we analyzed the variability of pads included in the DF/PF stimulation patterns during 20 daily sessions in 10 stroke patients. We also tracked the changes in the patients’ active and FES-induced ankle range of motion to quantitatively assess the effectiveness of the proposed FES methodology for inducing good-quality movement.

## Methods

### Patients

Ten hemiplegic patients (6 male and 4 female, aged 47–68 years.) with foot drop caused by stroke participated in this study. Table 1 shows the demographic and clinical data for all the participants. The experimental procedures and potential risks were explained to each patient individually, and each patient provided written consent. Ethical approval for the study was obtained from the local ethics committee. The inclusion criteria were: foot drop due to a stroke, adequate cognitive and communication skills to provide informed consent, and a sufficient passive ankle range of motion in all directions from the neutral (plantigrade) position.

**Table 1:** Demographic and clinical data of the 10 patients

Patien ID	Sex/age	Months since onset	Affected side	Stroke diagnosis	FM	BI	BBS	MAS	Aid
1	M/56	3	Right	Hemo	67	90	32	1	QC + AFO
2	F/63	3	Right	Isch	61	80	31	0	TC + AFO
3	M/54	5	Right	Isch	63	80	54	1	TC + AFO
4	F/58	6	Right	Isch	65	80	44	1	SC + AFO
5	M/66	7	Right	Isch	74	85	50	0	CS + AFO
6	M/62	12	Right	Hemo	60	55	34	1	QC + AFO
7	M/68	16	Right	Isch	53	90	35	2	AFO
8	F/47	24	Right	Hemo	45	85	36	2	SC + AFO
9	F/50	60	Left	Isch	67	90	50	0	SC + AFO
10	F/65	96	Left	Isch	68	85	43	1+	SC + AFO

*Abbreviations*: *Stroke diagnosis*: ischemic (*Isch*), hemorrhagic (*Hemo*), *FM* The Fugl-Meyer Test - motor and sensory impairment, *BI* The Barthel Index - assessment of daily activity impairment, *BBS* The Berg Balance Scale - static and dynamic balance abilities, *MAS* The Modified Ashworth Scale - measure of spasticity, *Aid* Ankle Foot Orthosis (*AFO*), simple cane (*SC*), three pod cane (*TC*), quad cane (*QC*)

### Hardware

The Fesia Walk system (Tecnalia R&I, Donostia/San Sebastián, Spain) was specifically designed and developed for multi-pad electrode applications. The Fesia Walk stimulator delivered a train of biphasic pulses of various widths and amplitudes to a demultiplexer, which routed them to different conductive pads of the electrode in an asynchronous manner. This operating principle (i.e., the surface-distributed low-frequency asynchronous stimulation - sDLFAS) has been investigated in our previous studies [30–32]. The integrated stimulator unit could be controlled with a PC, a tablet or a mobile phone via a user-friendly graphical interface. The stimulator output was current-controlled in steps of 1 mA and limited to 50 mA.

**Fig. 1:**
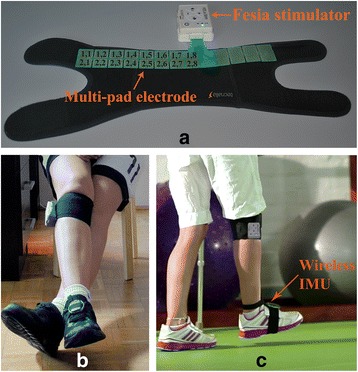
Fesia Walk system (Tecnalia R&I, Donostia/San Sebastián, Spain). **a** Electrical stimulator and multi-pad electrode with physical coordinates attached to the garment. **b** Position of a patient during setup process. **c** FES-assisted gait

A wireless inertial measurement unit (IMU), which comprises a MEMS accelerometer and gyroscope in a single chip (MPU-6050), was used to measure foot movements. The IMU was placed on the inset of the foot and attached with a buckle, allowing for secure and easy fastening to the patient’s foot (Fig. 1). Communication between the IMU and stimulator unit was based on the ZigBee protocol.

The custom-made multi-pad electrode consisted of 16 rectangular conductive pads that acted as cathodes and 4 pads that acted as anodes. This design enabled the coverage of two regions of interest for producing PF and DF, one over the tibial nerve and the other over the common peroneal nerve, targeting the tibialis anterior, gastrocnemius, peroneus, and soleus muscles. The pads were covered with an adhesive conductive gel to achieve an adequate skin-electrode interface (AG735, Axelgaard, Inc. [33]). The multi-pad electrode was attached to the garment and positioned in the popliteal fossa (behind the knee). The Fesia Walk system is shown in Fig. 1.

### Decision support system

The DSS was designed to find the optimal combination of the pad location and the stimulation intensity within the multi-pad electrode for electrically inducing four movements of the paretic foot: DF, PF, EV and IV. The DSS output provided four suggested pad-amplitude combinations for each of the four movements. The pad-amplitude suggestions for each movement group were ranked based on a newly introduced quantitative measurement of the quality of the induced movement designated the Q factor. Four quality ranks, Q1-Q4, were defined, where Q1 indicated the best quality. For example, Q1(DF) was the pad-amplitude combination with the highest estimated quality of the four suggestions for inducing a DF movement.

**Fig. 2:**
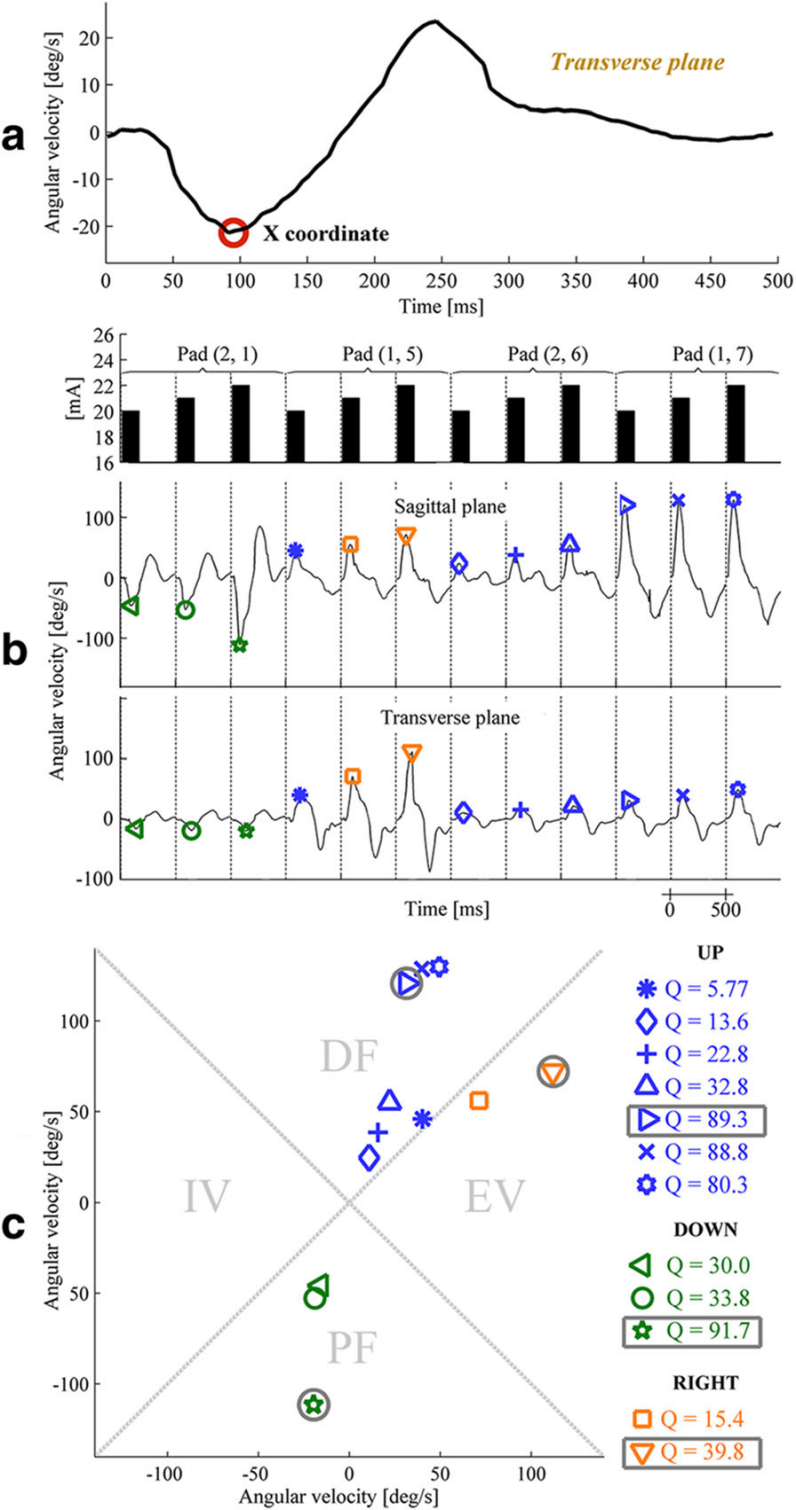
**a** Determination of the transverse plane peak - X. Zero on the time axis marks the stimulus onset, and X was determined as the global extreme with a shorter latency to the stimulus. **b** Three graphs showing representative 12 epochs (4 pads × 3 current amplitudes) of the twitch protocol (*vertical dotted lines* separate the individual twitch epochs) from one twitch protocol of one patient. The top panel shows the stimulus trains, with *black bars* marking the individual train timing, duration and intensity. The middle and bottom panels show the foot angular velocities in the sagittal and transverse planes, respectively. The X and Y peaks are marked with different symbols for each twitch epoch. *Blue symbols* mark the movements classified as UP, green - DOWN and orange - RIGHT. **c** *Bottom panel* shows the estimated twitch points (X, Y) in a 2D coordinate system. The *symbols* and *color-coding* correspond to those from (**b**). The points with the highest Q factors are *circled* with a *black line*. For selected representative set epochs, none of the twitches was classified as LEFT (i.e., IV)

Shaping an optimal stimulation electrode implies the necessity to evaluate the movement generated by each possible pad-amplitude combination. We designed a twitch protocol for this purpose, and the muscle twitches elicited in response to a short stimulation pulse train were classified into groups of different movement types (in this case, four groups - DF, PF, EV, and IV). The twitches were recorded with the IMU in the form of angular velocity signals in the sagittal and transverse planes. Each electrode pad was activated by 3 trains of stimulation pulses with pulse amplitudes that increased in 1 mA steps. Stimulus trains consisted of seven pulses with a frequency of 40 Hz and a pulse width of 400 μs (total train duration: 150 ms). A relaxation period of 350 ms was allowed between 2 consecutive stimulus trains, and thus, the time window for registering a twitch response for one pad-amplitude combination was 500 ms. Consequently, 48 twitch responses (16 pads × 3 current amplitudes, 24 s duration were recorded) in each of the two planes. The timing, duration and current intensity of the stimulus trains are presented in the top panel of Fig. 2 (b).

The initial stimulus train amplitudes were set above the motor threshold and determined manually for each patient, guided by previous experience, sensitivity to stimulation reported by the patients themselves (to avoid an unpleasant or painful sensation) and the observed foot responses to test stimuli. The current amplitudes ranged from 16 to 25 mA for all patients.

To extract the individual twitch responses, 2 continuous angular velocity signals in the sagittal and transverse planes were segmented into 0.5 s epochs, each starting with the stimulation train onset. The signal local extreme (maximum/minimum) with the shortest latency from the stimulus onset was found in both planes (i.e., the transverse plane peak was X, and the sagittal plane peak was Y). An example of the determination of X is shown in Fig. 2 (a). The bottom panel of Fig. 2. (b) shows representative raw gyroscope data in the sagittal and transverse planes from a single session with one patient. The X and Y values for the twitch movements associated with the four representative pads and 3 current amplitudes (for each pad) are marked with different colored symbols.

Each twitch epoch was translated into a point with (X, Y) coordinates in a 2D coordinate system, in which the x- and y-axes represented the angular velocities in the transverse and the sagittal planes, respectively (Fig. 2 (c)). The resulting twitch for each pad-amplitude combination was classified into one of the 4 movement groups (“UP”, “DOWN”, “LEFT”, “RIGHT”), and the Q factor was calculated. Classification of the twitch response and the calculation of the Q factor were completed as follows:

- ˝UP˝:    |Y| ≥ |X| and Y ≥ 0; Q = |Y|-|X|
- ˝DOWN˝:  |Y| > |X| and Y < 0; Q = |Y|-|X|
- ˝LEFT˝:   |X| ≥ |Y| and X ≤ 0; Q = |X|-|Y|
- ˝RIGHT˝:  |X| > |Y| and X > 0; Q = |X|-|Y|

Figure 2 (c) shows the division of the coordinate system into four classification areas; the corresponding symbols from Fig. 2 (b) were used to indicate the coordinates of the points in Fig. 2 (c). The “UP” and “DOWN” segments of the coordinate system corresponded to the DF and PF movement groups, respectively. If the right leg was paretic, the “LEFT” and “RIGHT” segments corresponded to IV and EV, respectively, whereas for the left leg, “LEFT” and “RIGHT” corresponded to EV and IV, respectively. When a single pad was assigned multiple Q factor values within the same movement group (each for a different pulse amplitude), only the pad-amplitude combination with the highest Q was retained. In each movement group, the top 4 ranked pad-amplitude combinations (Q1-Q4) were suggested to the user for further stimulation pattern design.

### Stimulation electrode shaping

The clinicians who participated in the present study were 2 medical doctors and 4 physical therapists who had previous experience with electrical stimulation. They underwent 2 weeks of training to become familiar with the new multi-pad concept of stimulation as well as the use of the PC application for stimulation electrode shaping. The patterns were shaped via a user-friendly application on a touch-screen tablet PC. For better localization, the selected pads were highlighted in the electrode sketch displayed on the screen. In most cases, at least 2 clinicians were present during therapy, with one operating the stimulation system and the others supervising the process. The final DF/PF patterns were approved by all clinicians present.

The clinicians were instructed to observe the foot responses during the twitch protocol and annotate the pads that induced satisfactory twitch responses in the DF and PF directions. The criterion for the DF or PF pad annotation was that the movement angle and direction were considered adequate for inclusion in the DF/PF stimulation patterns. The clinicians’ task was to annotate at least one useful pad per DF and PF directions during the twitch protocol. The pad locations and current amplitudes were displayed on the screen of the tablet PC during the twitch protocol so the clinicians could easily identify and write down their choice of pads for both movement directions. After the twitch protocol was completed, the clinicians proceeded with the final stimulation electrode shaping using the combined information of their annotated choices of satisfactory pads and the DSS output. The following abbreviations were introduced to improve readability:

1. Clinicians’ choice of pads producing satisfactory movements during the twitch protocol for DF and PF were designated Set(DF)_TW_ and Set(PF)_TW_, respectively.
2. The sets of DF and PF pads suggested by the DSS were designated Set(DF)_DSS_ and Set(PF)_DSS_, respectively.
3. The sets of pads included in the final DF and PF patterns shaped by the clinicians and used for FES-assisted walking were designated Set(DF)_FIN_ and Set(PF)_FIN_, respectively.

To explain the pattern-shaping process, we used the DF movement as an example, and the same set of rules was applied for PF. The first step was to determine the intersection between Set(DF)_TW_ and Set(DF)_DSS_ and test the highest Q-rated pad-amplitude of the intersection with a 2-s tetanic FES contraction. When adding a new DF pad to the pattern, the pads that belonged to the Set(DF)_TW_ and Set(DF)_DSS_ intersection were given priority, and the inclusion order was determined by the Q-rank (from the highest rank downward). If a pad had to be included and pads from the intersection were used/non-available, the remaining pads from the Set(DF)_TW_ were considered for inclusion in a random order. The clinicians proceeded to search for an optimal combination using the remaining pads from Set(DF)_DSS_, Set(EV)_DSS_, Set(IV)_DSS_ or non-suggested pads only after all pads from the Set(DF)_TW_ were tested. Therefore, the choice of pads shaping the electrode was primarily guided by the clinicians’ visual inspection of the twitch protocol and their choice of a satisfactory set of DF/PF pads. Only the order of pad inclusion in the final pattern was guided by the DSS output (i.e., was ordered by the Q-rank of the pads from the intersection of Set(DF/PF)_TW_ and Set(DF/PF)_DSS_). Therefore, the selection bias introduced by the presence of the DSS output during the electrode shaping process is reflected mainly in the order of the pads tested, where the highest priority was assigned to the pads identified both by the clinicians and the DSS in descending order. However, if the clinicians’ choice of useful pads and the DSS output did not agree, the clinicians were instructed to first test their choices in random order before proceeding to the DSS-suggested pads and/or non-suggested pads to decrease the previously mentioned bias introduced by the DSS output.

When a new pad was considered for inclusion in the pattern, its effect alone was checked as well as its contribution to the existing pattern. If the contribution of a newly considered pad was insufficient, it was omitted from the pattern. Furthermore, if a newly added pad provided better movement alone, the previously added pads were excluded from the pattern. The addition of a new pad to the pattern was based on the direction and amplitude of the resulting movements in the 2-s tetanic test, and the clinicians were guided by the following set of rules:

I. If the direction and amplitude of the FES-induced movement were satisfactory, the current pattern was stored as the final pattern.

II. If direction was satisfactory but amplitude was not, the first step was to increase the current intensity until the movement amplitude was adequate. If an increase of 3–4 mA did not provide adequate movement, a new pad was considered for electrode shaping.

III. If direction was not satisfactory (i.e., an overly pronounced EV or IV was present), a new pad was considered. This could result in either the exclusion of the previous pads or the correction of the movement direction (by the contribution of a new pad to contrasting movement direction).

This procedure was introduced as an expert evaluation tool of the DSS performance.

At any moment, an unpleasant sensation reported by the patient led to a decrease in the current amplitude or the omission of the last-added pad. There were no limitations in the maximum number of pads, but only pads that contributed to the quality of the movement were included in the final patterns for DF and PF.

### Protocol

The DSS testing was performed in the Clinic for Rehabilitation ˝Dr. Miroslav Zotović˝ in Belgrade, Serbia. A clinician placed a garment with a multi-pad electrode and stimulation unit around the patient’s knee and the IMU sensor on the foot. The multi-pad electrode was placed over the lateral and medial popliteal fossa, and the pad with the coordinates (2, 7) was positioned on the head of the fibula (shown in Fig. 1). Three (of four) neighboring pads of the multi-pad anode were selected according to the lower leg circumference such that the middle pad was positioned below the patella. All patients were seated during the tests. The healthy leg was fully extended without medial or lateral rotation and with the heel touching the ground and the foot in relaxed position. The paretic leg was positioned over the healthy one, crossing it at approximately knee level (Fig. 1 (b)). The paretic leg knee angle was approximately 160 degrees, and the foot hung freely.

First, the active range of motion (ROMa) was recorded with the IMU. This task involved the patient independently lifting and lowering the paretic foot, guided by a prerecorded voice command sequence (voice command: ‘Up’ at the beginning, ‘Down’ after 5 s and a beep sound at the end of the sequence). The estimation of the foot range of motion (ROM) was performed under pseudo-static measurement conditions: the foot was stationary before the abrupt movements in DF and PF directions, with a relatively short transitions to the angle plateaus in which the foot was sustained. With this in mind, only the accelerometer signals were used to estimate the foot angles in static periods. The foot tilt angles were estimated based on the gravitational component of the acceleration and calculated as the arctangent of the ratio of the acceleration values in transverse and sagittal planes according to the method described in [34]. Estimation of the foot ROM relies on 3 plateaus: the first one was estimated before the reaction to the ˝Up˝ command, and the second and third were related to the maximal DF and PF, respectively. Finally, the foot ROM was calculated as the difference between the medians of the second and third plateaus.

The next step in the setup procedure was to choose the current amplitude range (e.g., 20–22 mA), followed by the twitch protocol. During the twitch protocol, if the patient reported that the pad-amplitude combination produced a painful sensation, it would be excluded from the DSS output. The automatic algorithm suggested pad-amplitude combinations for DF, PF, EV and IV, and the clinicians formed the final DF/PF patterns. The foot trajectories elicited by the electrical stimulation for calculating the stimulated ROM (ROMs) were acquired by activating the final DF pattern with a 5 s duration, followed by the activation of the final PF pattern for 7 s. The ROMs was calculated in the same manner as for the ROMa. The ROMs was used as an objective indicator of quality of the pattern chosen.

Each patient underwent the twitch protocol five times a week for four weeks and were supervised by the clinicians. The clinicians could overrule the suggested combinations. Following the setup process, which took up to 5 min, the patients received 30 min of FES-assisted walking therapy using the final selected stimulation pads and amplitudes. The frequency of the stimulation was set at 40 Hz and pulse width at 400 μs during the entire setup process as well as during the FES-assisted gait.

The triggering of the stimulation during the FES-assisted gait was automated. The final patterns were stored in the stimulator memory, and the triggering of the stimulation was independent of the tablet PC. The detection of the gait phases was based on the signal in the sagittal plane from the IMU gyroscope. The pattern for PF was activated in the push-off phase, right after the initial heel lifting. Before the toes lifted, the pattern for PF was deactivated, and the pattern for DF was activated because a muscle contraction is delayed after the onset of stimulation. The DF pattern was active during the entire swing phase of the gait. Thus, firm foot lowering (heel contact – toes contact) could occur, and the stimulation was turned off after the heel contact and forward propagation of the body. The outcomes of the FES-assisted gait therapy with the Fesia Walk system are not discussed in this manuscript except to demonstrate that there was an improvement in the ankle ROM over time using the FES-assisted gait therapy.

### Statistical analysis

Statistical analyses were conducted to assess the following effects:

- The differences between ROMa and ROMs within the same session (intra-session ROM differences) and
- The differences in ROMa or ROMs over time, i.e., over different sessions (inter-session ROM differences)

The Kolmogorov–Smirnov test was applied to test the normality of the ROMa/ROMs data. A Friedman two-way analysis of variance by ranks test was used to evaluate the inter-session ROM differences. If the Friedman test revealed significant differences, a post hoc analysis with Wilcoxon’s signed rank test was used to compare the ROMa/ROMs of the baseline sessions with the ROMa/ROMs of sessions 2–20. The Bonferroni correction for multiple comparisons was used to determine the significance threshold: 0.05/19 = 0.0026. Moreover, Wilcoxon’s signed rank test was used to evaluate the intra-session ROM differences with the significance threshold set at 0.05.

## Results

**Fig. 3:**
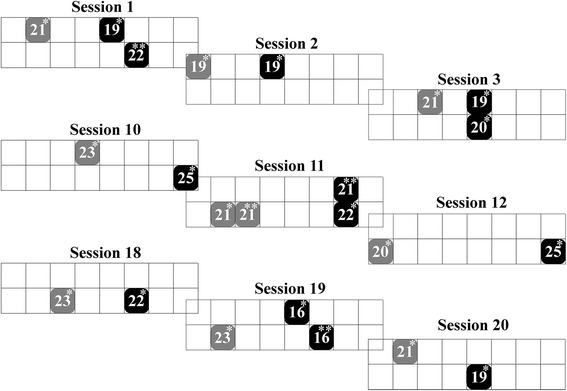
Chosen pad (Set_FIN_) allocation and current intensities for DF (*black pads*) and PF (*gray pads*) for the first three, middle three and last three sessions of patient 8. Pads with one asterisk in the *upper right corner* are the top-ranked pads (Q1) by DSS, and those with 2 asterisks are the 2nd-ranked pads (Q2) by DSS

The efficacy of the DSS for finding the best subset of pads for inducing DF and PF was evaluated by comparing the set of pads suggested by the DSS (Set_DSS_) and the final set of pads that constituted the stimulation pattern selected by the clinicians (Set_FIN_). Because the clinicians had the freedom to choose any combination of the Q-rated (suggested) as well as non-suggested pads when they made their final decision, we reviewed the Q-values of the pads included in every Set_FIN_ for inducing the two movements over all the therapy sessions. The results are summarized in the pie charts shown in Fig. 3 (a) for DF and (b) for PF. In Fig. 3, the final stimulation patterns for DF and PF are grouped into three categories based on the Q-rank of the pads included:

I. Set(DF)_FIN_ and Set(PF)_FIN_ included only Q-rated pads of the corresponding movement group (DF or PF, respectively) - gray slices.
II. Set(DF)_FIN_ and Set(PF)_FIN_ included only Q-rated pads of the corresponding movement group with additional Q-rated pads of EV and IV groups - yellow slices.
III. Set(DF)_FIN_ or Set(PF)_FIN_ included any of the non-suggested pads - white slices.

The size of each slice shows the inclusion percentage of a unique rank-dependent combination of pads over 200 therapy sessions. The term rank-dependent combination is used to describe a pattern defined by the Q factors of the included pads independent of their physical coordinates within the array electrode. For example, in session 1 of patient 8, Set(DF)_FIN_ comprised a Q1 pad with the physical coordinates (2,5) and a Q2 pad with coordinates (1,6) (depicted in Fig. 3; Fig. 1 (a) provides the physical coordinates of the pads for comparison). For session 11 with the same patient, Set(DF)_FIN_ again comprised Q1 and Q2, but in this case, the physical coordinates of those pads within the array were (2,7) and (1,7), respectively. This example illustrates that the same rank-dependent combination (Q1Q2) can be composed of various sets of physical pads for different sessions.

Figure 3 shows the Set(DF)_FIN_ and Set(PF)_FIN_ allocations and selected current intensities in the first three, the middle three and the last three sessions for one representative patient. The pad positions, pad numbers and current amplitudes change in successive sessions. The data presented in Fig. 3 show two effects. First, large variations were present in the pad locations and numbers of included pads from session to session. Second, the individual pads within Set(DF)_FIN_ were mainly located in the right portion of the electrode and Set(PF)_FIN_ in the left portion of the electrode.

During all therapy sessions, clinicians chose 22 different rank-combinations for DF and 20 for PF, but only 5 combinations for DF and 2 combinations for PF occurred in more than 5% of sessions. Two of the most frequent combinations for DF were Q1Q2 in 33.5% and Q1 in 19%, whereas those for PF were Q1 in 52.5% and Q1Q2 in 19.5%. The combination Q1(DF)Q1(EV) was included in 7% of the DF patterns.

The average number of pads included in all DF patterns was 2.26 (±0.9) and 1.71 (±0.7) for PF patterns. The maximum number of pads included in the patterns made by clinicians showed that more than four stimulation pads were included only in 2 sessions (5 pads each), which justifies our hypothesis that offering four Q-ranked pads per movement would be sufficient for shaping a stimulation electrode.

**Fig. 4:**
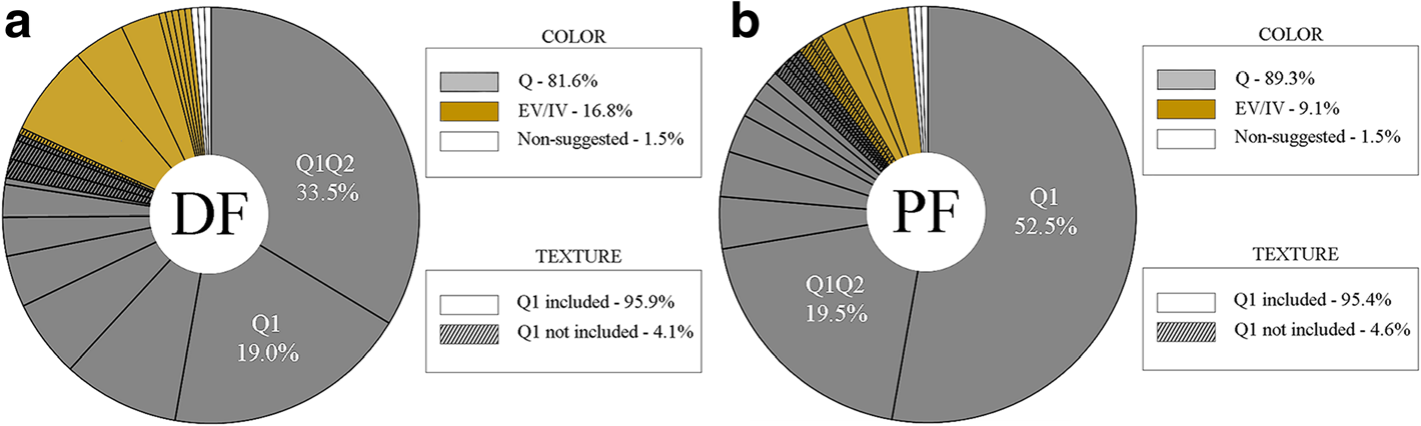
Pie charts of all patterns for DF (**a**) and PF (**b**). *Gray slices* represent the patterns comprising pads suggested by DSS, *yellow slices* are the patterns including at least one pad for EV or IV, and *white slices* are the patterns containing a non-suggested pad. Patterns not including the top-ranked pad (Q1) are hatched

More than 95% of the time, the clinicians included the top-ranked (Q1) pad (95.9% for DF and 95.4% for PF), which is also marked in the chart in Fig. 4 by including the texture (see captions for explanations). For both movements, patterns comprising the Q1 or Q1Q2 pads were used in more than 50% of the sessions for DF and in more than 70% of the sessions for PF. The results also demonstrate that in 82% and 89% of the sessions, the clinicians included only the suggested pads for DF and PF, respectively. Moreover, the clinicians chose non-suggested pads only in 1.5% of the sessions, indicating that the Q-ranked pads were typically sufficient (98.5% of the cases) for creating good-quality stimulation patterns.

Importantly, clinicians included Q-ranked pads for EV or IV in their patterns 16.8% of the time for DF and 9.1% for PF (indicated by the total size of the yellow slices per the chart in Fig. 4). The inclusion of EV was desirable in some cases, as individuals with foot drop often tend to invert the foot during the swing phase of the gait and land on the lateral side of the foot. Additional eversion increases the ankle stability and weight acceptance [7].

To analyze the inter-session Set_FIN_ variability for each patient, we introduced an electrode coordinate system in which the electrode pads were represented in a two-dimensional plane, with the x-axis in line with the row and the y-axis in line with the column of the multi-pad electrode (Fig. 1 (a)). The electrode presented in this coordinate system consisted of orthogonal pads; the distance between the centers of two neighboring pads was 1, and the distance between the centers of two neighboring diagonal pads was √2.

**Fig. 5:**
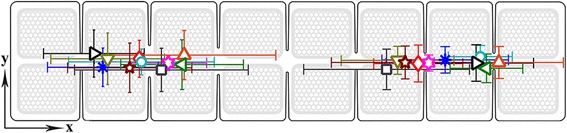
Electrode coordinate system with coordinates of 10 patients’ global mean pads and associated standard deviations, marked with different symbols

We calculated the *global mean pad* and *session mean pad* coordinates (x, y) for DF and PF separately for each patient. The session mean pad coordinates were derived as the arithmetic mean of the (x, y) coordinates of all the pads included in Set_FIN_ in a single session. The global mean pad coordinates were calculated as the arithmetic mean of all session mean pads for each patient. Fig. 5 shows the global mean pads with different symbols/colors for each patient and the standard deviations of the distances between the global mean pads and session mean pads (i.e., the error bars in the x and y directions), plotted over the electrode layout. The global mean pad coordinates and associated standard deviations are given in Table 2 (˝Mean pad˝ section). Table 2 also contains the physical coordinates of pads included in most Sets_FIN_ for DF/PF (most frequent pads - MFPs) and the number of patterns that included the most frequent pad (nP).

**Table 2:**
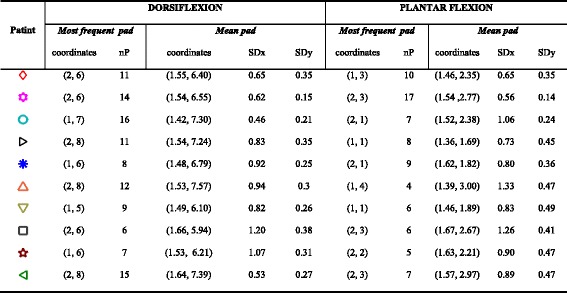
Position and variability of the chosen pads for dorsiflexion and plantar flexion

*Abbreviations*: Most frequent pad: x and y coordinates of MFP within the multi-pad electrode, nP – the number of patterns which contain MFP. Mean pad: mean x and y coordinates of global mean pad for patients within the multi-pad electrode, SDx and SDy –standard deviation of session mean pads in horizontal and vertical axes of the electrode. Symbols in the Patient column mark the locations of global mean pads in Fig. 5

**Fig. 6:**
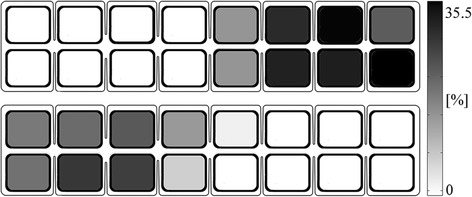
Percentage of pad inclusions in the final patterns for DF (*upper panel*) and PF (*lower panel*) in 200 sessions (all patients and all sessions)

An analysis of the pads included in Set_FIN_ for all patients in all sessions indicates two primary effects: global, inter-patient grouping of Set(DF)_FIN_ and Set(PF)_FIN_ into two spatially distinct zones of the multi-pad electrode (shown in Fig. 6) and a pronounced inter-session variability for the pads included in Set(DF)_FIN_ and Set(PF)_FIN_ for each patient (Fig. 5).

**Fig. 7:**
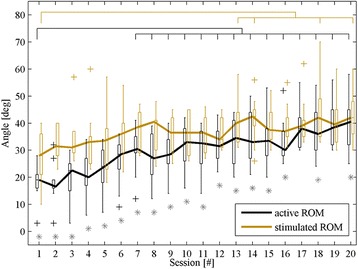
ROMa (*black*) and ROMs (*yellow*) values presented in boxplots. Lines connect the median values (*in degrees*) for all patients in 20 sessions. *Gray asterisks* represent the inter-session significant differences between ROMa and ROMs. *Horizontal bars* denote significant differences between the first session and those sessions marked with *vertical* ticks for ROMa (*black*) and ROMs (*yellow*)

Aside from addressing the DSS efficacy, a quantitative assessment of the FES therapy effect on ROMa and ROMs was performed. A Friedman analysis of the inter-session ROM differences revealed significant improvements in both ROMa and ROMs over time. A post hoc analysis using a Wilcoxon signed rank test revealed a significant improvement in ROMa starting after one week of FES-assisted walking therapy (i.e., the difference between session 1 and sessions 6–20, *p* < 0.05). The median baseline value (and interquartile range) of ROMa for all patients was 19 (16–21)°, whereas it was 28.5 (23–32)° after one week (i.e., session 6), and it was 40.5 (32–45)° at the end of therapy. Intra-session differences between ROMa and ROMs analyzed with the Wilcoxon signed rank test revealed significantly greater ROMs values for all sessions, except 17 and 19. The significantly larger values of ROMs compared to ROMa in the majority of sessions indicate that the stimulation patterns were adequately selected and facilitated the impaired movements. A lack of significant differences during the last days of therapy (sessions 17 and 19) can be attributed to the therapeutic effect reflected in ROMa. The differences between ROMs and ROMa (the convergence of ROMa and ROMs was an indication of therapeutic success) were reduced throughout the therapy due to a steeper increase in ROMa (Fig. 7). The difference between ROMa and ROMs during the first therapy sessions was approximately 10°, whereas the difference was less than 5° during the last session.

## Discussion

We have investigated the efficacy of a novel DSS for defining optimal sets of stimulation pads for producing both DF and PF movements in stroke patients using the Fesia Walk system (Tecnalia R&I, Donostia/San Sebastián, Spain). The results concerning pads included in the DF and PF patterns showed a global inter-patient spatial grouping of Set(DF)_FIN_ and Set(PF)_FIN._ This effect is reflected in the results presented in Fig. 6, showing the percentages of the pad inclusions in the final DF and PF patterns for all patients and sessions. The most probable surface areas of custom multi-pad electrodes identified showed that the electrode zones for activating DF and PF are clearly separated. The majority of the selected pads were concentrated in the 3 × 2 pad sections, which corresponded to a 5.70 × 3.70 cm area. When derived for individual patients, the global mean pads were all distributed within 1.6 pad sizes or 2.72 cm for DF and 1.3 pad sizes or 2.21 cm for PF, indicating relatively little inter-patient variability of the optimal stimulation areas for DF and PF induction. This grouping is in accordance with the underlying anatomy. The majority of the pads included in Set(PF)_FIN_ were located in the half of the electrode positioned over the medial popliteal area and targeted the tibial branch of the sciatic nerve (i.e., the left half of the electrode in Fig. 6). The majority of pads included in Set(DF)_FIN_ were in the half of the electrode that targeted the common peroneal nerve, passing through the lateral aspect of the popliteal fossa (i.e., the right half of the electrode in Fig. 6) [35].

Another important outcome of this study is the analysis of the inter-session variability of pads included in Set(DF)_FIN_ and Set(PF)_FIN_ for each patient. The possible sources of the observed effect may be attributed to slight differences in garment donning from session to session, changes in skin-electrode impedance and/or differences in the shank circumference due to swelling or changes in tissue hydration. All sources of variability listed cannot be avoided in realistic clinical or daily life applications. These results support the necessity of employing multi-pad electrodes. Their major advantage over conventional electrodes is the ability to shape the stimulation area in an optimal manner without precise positioning, as the group of pads covers a larger area of interest, which could also indicate why commercially available foot drop systems with a single pair of electrodes are not able to provide reliable ankle dorsiflexion at all times.

Our methodology was aimed at producing an adaptable stimulation pattern shape while retaining relatively small individual pad areas that provided increased selectivity of stimulation [20, 31]. However, a group of smaller pads within the array increases the number of the possible combinations for designing a stimulation pattern. Testing all of these combinations is time consuming and exhausting for both clients and clinicians, which leads to the need for a fully automated algorithm; the DSS described in this study is the first step toward such a goal. The fact that the final pads included formed two spatially distinct areas provides an opportunity to preselect certain pads for DF and PF and further reduce the search-zone per movement type. The results presented in Table 2 indicate that 5 different pads were most frequently identified for DF electrode shaping, and those pads were used in more than 50% of the sessions (>10) in 6 patients. For PF electrode shaping, 6 different pads were most frequently identified, and the most frequent pads were used in 50% sessions or more only in 2 patients. This result further confirms the variability of optimal stimulation hotspots within the DF and PF zones and suggests that an additional closed-loop motion-analysis-based refinement is necessary as a part of an automatic algorithm for electrode shaping.

The combined effect of the activation of several pads was not systematically evaluated during the clinical study presented here. However, in our previous studies, we examined certain effects related to an asynchronous multi-pad FES [12, 32]. The important factor that affects the summation effect was the choice of stimulation parameters. We selected a relatively high stimulation frequency (40 Hz) coupled with a short inter-pulse interval (IPI = 1 ms) to produce a responsive and strong muscle contraction suitable for walking (i.e., a strong push-off and fast foot clearance in the swing phase). The asynchronous FES regime implies that the burst of number (N) of consecutive pulses is routed to N pads. Therefore, an IPI value of 1 ms is the interval between the activation of consecutive pads within the multi-pad-shaped electrode. The 40 Hz stimulation frequency implies that the interval between the pulses sent to the same pad within the shaped electrode is 1/40 s (25 ms). A short IPI was selected to fit all the stimulation pulses in the nerve refractory period, preventing the consecutive activation of the same muscle fibers by stimulation pulses delivered to different electrode pads, thus reducing muscle fatigue, which occurs more rapidly from stimulation with high frequencies. This setup also affects the summation effect of a stimulation electrode that comprises several pads during asynchronous multi-pad stimulation. Due to the short IPI, a stimulus routed to a pad will only activate muscle fibers that were not activated by preceding stimuli, which means that the muscle force resulting from a combination of pads could not be exactly estimated after the single pad activations during the twitch protocol. Nevertheless, activating a combination of several pads from the same group (e.g., for DF) always produced movement in the selected direction, proving that there was no electrical current superposition that could lead to the activation of nerves not activated during twitch protocol.

Prenton et al. reported that a take-home array-based functional electrical stimulation system with automated setup is feasible for patients with foot drop [27]. However, the efficacy of this algorithm for producing satisfactory movement was not reported. The cathode positioning and anode placement over the tibialis muscle in this design were intended solely for DF and EV/IV control and prevented the induction of PF movement. To the best of our knowledge, our system is the first to support both DF and PF movements. The system enables the control of DF in the swing phase and PF in the push-off phase during FES-assisted walking using a single custom-designed multi-pad electrode and a single IMU.

A seated position was selected for the patient during the twitch protocol and electrode shaping process, as it was the safest and most comfortable and allowed for a minimally obstructed range of both DF and PF. Moreover, the position of the stimulated structures of the paretic leg during the twitch protocol was similar to that in which the DF and PF stimulation electrodes are triggered during the FES-assisted gait cycle. This was important for the successful translation of the DF and PF electrodes from the static condition during the twitch protocol to a dynamic condition during FES-assisted walking without the introduction of changes due to the relative displacement of the stimulated structures (i.e., underlying nerve branches and tissue) caused by a change in position from seated to standing. The adhesive properties of the electrode hydrogel ensured stable contact between the skin and electrode, and the electrode garment also secured the electrode position by fixing it to the stimulation site.

Although variability of the optimal stimulation sites for daily sessions was observed and reported in other publications [21, 26], this effect was never systematically evaluated. Furthermore, this study is the first to use an automated, quantitative assessment of the stimulated ROM to validate a qualitative pattern assessment performed by the clinicians. In more than 95% of cases, the clinicians were satisfied with the effect of the top-ranked pad activations. To further strengthen and accelerate muscle contraction and/or to produce ankle flexion with pronounced eversion, which is a desirable movement during rehabilitation [7], the clinicians introduced additional pads to the proposed stimulation patterns until the foot movement was considered adequate. The results also demonstrate that in 82% and 89% of all sessions, the clinicians included only the pads suggested by the DSS; i.e., the Set(DF)_FIN_ and Set(PF)_FIN_ were subsets of Set(DF)_DSS_ and Set(PF)_DSS_, respectively. This result indicates that the pads suggested by the automatic algorithm were typically sufficient for creating good-quality stimulation patterns for both movements. Moreover, Set(DF)_FIN_/Set(PF)_FIN_ were subsets of Set(DF)_DSS_/Set(PF)_DSS_ plus Set(EV)_DSS_/Set(IV)_DSS_ in both movements for 98.5% of the trials. These results demonstrate that the DSS was successful in offering adequate choices for the construction of stimulation patterns for DF and PF movements, thus reducing the number of possible choices and facilitating the decision-making process of pad selection, which was the main purpose of the DSS presented in this study.

The quality of the chosen patterns was validated by the response of the shank muscle activations with electrical stimulation (ROMs). Quantitative validation of the final selected stimulation patterns indicated that they did indeed produce the desired movements. Aside from the ROMs, we tracked the ability of a person to voluntarily flex and extend their ankle (ROMa). We observed an assistive effect and a therapeutic effect of foot drop walking therapy with the selected patterns. The assistive effect (i.e., a ROMs that is significantly greater than ROMa, *p* < 0.01) was present with the first use of the system, but it decreased in later stages of therapy due to the therapeutic effect on the foot ROMa (Fig. 7). Compared to baseline, ROMa increased significantly starting after one week of therapy (session 6, *p* < 0.0026), even though more than 3 months had passed since the occurrence of stroke. ROMs showed a statistically significant increase at the end of the study compared to the baseline, possibly due to an increase in muscle strength and a reduction in spasticity (*p* < 0.0026). The median value of the ROMs for all patients in the last session was 42 [Q1–Q3: 36–48]°, whereas it was 28 [Q1–Q3: 21–36]° in the first session. Further investigation is necessary to determine whether the therapeutic effects persist during a follow-up period. The fact that the stimulated ROM across the majority of sessions was significantly higher (*p* < 0.05) than the matching voluntary ROM demonstrates that the assistive potential of this type of stimulation remains observable regardless of a patient’s individual recovery pattern.

## Conclusions

We have described a new DSS for finding an optimal set of pads to produce both DF and PF movements. We demonstrated that a single custom-designed multi-pad electrode can selectively induce both DF and PF movements. The control of both DF and PF movements and the stimulation hotspots differed from all previously reported methodologies used for foot drop correction. The pronounced session-to-session variability of the stimulation patterns emphasizes the advantages of multi-pad electrodes and the need for automation to facilitate stimulation pattern design. The semi-automatic procedure of statistical analysis of Q-ranked combinations and inclusion data on the final patterns optimized by the clinicians can serve as an initial step in this direction. Future work will address the effectiveness of the gait therapy using the Fesia Walk system and the design of a fully automated electrode shaping algorithm.

## Abbreviations

AFO: Ankle Foot Orthosis
BBS: The Berg Balance Scale
BI: The Barthel Index
DF: Dorsiflexion
DSS: Decision support system
EV: Eversion
FES: Functional electrical stimulation
FM: The Fugl-Meyer Test
Hemo: Hemorrhagic stroke
IMU: Inertial measurement unit
Isch: Ischemic stroke
IV: Inversion
MAS: The Modified Ashworth Scale
MFP: Most frequent pad
nP: The number of patterns which contain MFP
PF: Plantar flexion
QC: Quad cane
ROM: Range of motion
SC: Simple cane
SDx: Standard deviation of sessions mean pads in horizontal plane of the electrode
SDy: Standard deviation of sessions mean pads in vertical plane of the electrode
Set(DF)_DSS_: Set of pads suggested for DF by the DSS
Set(DF)_FIN_: Set of pads included in final pattern for DF by clinicians
Set(DF)_TW_: Clinicians’ choice of pads producing satisfactory movements during the twitch protocol for DF
Set(PF)_DSS_: Set of pads suggested for PF by the DSS
Set(PF)_FIN_: Set of pads included in final pattern for PF by clinicians
Set(PF)_TW_: Clinicians’ choice of pads producing satisfactory movements during the twitch protocol for PF
TC: Three pod cane

## References

[1] Burridge J, Taylor P, Hagan S, Wood D, Swain I (1997). The effect of common peroneal nerve stimulation on quadriceps spasticity in hemiplegia. Physiotherapy.

[2] Laufer Y, Hausdorff JM, Ring H (2009). Effects of a foot drop neuroprosthesis on functional abilities, social participation, and gait velocity. Am J Phys Med Rehabil.

[3] Bajd T, Kralj A, Štefančič M, Lavrač N (1999). Use of functional electrical stimulation in the lower extremities of incomplete spinal cord injured patients. Artif Organs.

[4] Laufer Y, Ring H, Sprecher E, Hausdorff JM (2009). Gait in individuals with chronic hemiparesis: one-year follow-up of the effects of a neuroprosthesis that ameliorates foot drop. J Neurol Phys Ther.

[5] Burridge J, Taylor P, Hagan S, Swain I (1997). Experience of clinical use of the Odstock DroppedFoot stimulator. Artif Organs.

[6] Bethoux F, Rogers HL, Nolan KJ, Abrams GM, Annaswamy TM, Brandstater M (2014). The effects of Peroneal nerve functional electrical stimulation versus ankle-foot Orthosis in patients with chronic stroke a randomized controlled trial. Neurorehabil Neural Repair.

[7] Stein RB, Everaert DG, Thompson AK, Chong SL, Whittaker M, Robertson J (2010). Long-term therapeutic and orthotic effects of a foot drop stimulator on walking performance in progressive and nonprogressive neurological disorders. Neurorehabil Neural Repair.

[8] Melo P, Silva M, Martins J, Newman D (2015). Technical developments of functional electrical stimulation to correct drop foot: sensing, actuation and control strategies. Clin Biomech.

[9] G. M. Lyons, T. Sinkjær, J. H. Burridge, and D. J. Wilcox, “A review of portable FES-based neural orthoses for the correction of drop foot,” *Neural Systems and Rehabilitation Engineering,* IEEE Transactions on*,* vol. 10, pp. 260-279, 2002. 10.1109/TNSRE.2002.806832 12611364

[10] Neptune R, Kautz S, Zajac F (2001). Contributions of the individual ankle plantar flexors to support, forward progression and swing initiation during walking. J Biomech..

[11] Burridge J, Taylor P, Hagan S, Wood DE, Swain ID (1997). The effects of common peroneal stimulation on the effort and speed of walking: a randomized controlled trial with chronic hemiplegic patients. Clin Rehabil.

[12] Malešević NM, Popović LZ, Schwirtlich L, Popović DB (2010). Distributed low-frequency functional electrical stimulation delays muscle fatigue compared to conventional stimulation. Muscle Nerve.

[13] Taylor P, Burridge J, Dunkerley A, Wood D, Norton J, Singleton C (1999). Clinical audit of 5 years provision of the Odstock dropped foot stimulator. Artif Organs.

[14] Valtin M, Seel T, Raisch J, Schauer T (2014). Iterative learning control of drop foot stimulation with array electrodes for selective muscle activation. IFAC Proceedings Volumes.

[15] Kenney LP, Heller BW, Barker AT, Reeves ML, Healey TJ, Good TR (2015). The design, development and evaluation of an array-based FES system with automated setup for the correction of drop foot. IFAC-PapersOnLine.

[16] Hausdorff JM, Ring H (2008). Effects of a new radio frequency–controlled neuroprosthesis on gait symmetry and rhythmicity in patients with chronic hemiparesis. Am J Phys Med Rehabil.

[17] Bijelić G, Popović-Bijelić A, Jorgovanović N, Bojanić D, Popović DB (2004). E Actitrode: the new selective stimulation interface for functional movements in hemiplegics patients. Serbian J Electrical Eng.

[18] Westerveld AJ, Schouten AC, Veltink PH, van der Kooij H (2012). Selectivity and resolution of surface electrical stimulation for grasp and release. IEEE Trans Neural Syst Rehabil Eng.

[19] T. Keller, M. Lawrence, A. Kuhn, and M. Morari, “New multi-channel transcutaneous electrical stimulation technology for rehabilitation,” In Engineering in Medicine and Biology Society*,* 2006*.* EMBS’06. 28th Annual International Conference of the IEEE, 2006, pp. 194-197. 10.1109/IEMBS.2006.259399 17946802

[20] Popović DB, Popović MB (2009). Automatic determination of the optimal shape of a surface electrode: selective stimulation. J Neurosci Methods.

[21] C. Azevedo-Coste, G. Bijelic, L. Schwirtlich, and D. B. Popovic, “Treating drop-foot in hemiplegics: the role of matrix electrode,” In 11th Mediterranean Conference on Medical and Biomedical Engineering and Computing 2007, 2007, pp. 654–657.

[22] Koutsou AD, Moreno JC, del Ama AJ, Rocon E, Pons JL (2016). Advances in selective activation of muscles for non-invasive motor neuroprostheses. J Neuroeng Rehabil.

[23] De Marchis C, Monteiro TS, Simon-Martinez C, Conforto S, Gharabaghi A (2016). Multi-contact functional electrical stimulation for hand opening: electrophysiologically driven identification of the optimal stimulation site. J Neuroeng Rehabil.

[24] A. Elsaify, “A self-optimising portable FES system using an electrode array and movement sensors,” Engineering, 2005.

[25] Heller BW, Clarke AJ, Good TR, Healey TJ, Nair S, Pratt EJ (2013). Automated setup of functional electrical stimulation for drop foot using a novel 64 channel prototype stimulator and electrode array: results from a gait-lab based study. Med Eng Phys.

[26] Kenney LP, Heller BW, Barker AT, Reeves ML, Healey J, Good TR, et al. A review of the design and clinical evaluation of the ShefStim array-based functional electrical stimulation system: Med Eng Phys; 2016. 10.1016/j.medengphy.2016.08.005 27639656

[27] Prenton S, Kenney LP, Stapleton C, Cooper G, Reeves ML, Heller BW (2014). Feasibility study of a take-home array-based functional electrical stimulation system with automated setup for current functional electrical stimulation users with foot-drop. Arch Phys Med Rehabil.

[28] Tan Z, Liu H, Yan T, Jin D, He X, Zheng X (2014). The effectiveness of functional electrical stimulation based on a normal gait pattern on subjects with early stroke: a randomized controlled trial. Biomed Res Int.

[29] Takeuchi N, Izumi S-I (2013). Rehabilitation with poststroke motor recovery: a review with a focus on neural plasticity. Stroke Res Treat.

[30] J. Malesevic, N. Malesevic, G. Bijelic, T. Keller, and L. Konstantinovic, “Multi-pad stimulation device for treating foot drop: Case study,” In Functional Electrical Stimulation Society Annual Conference (IFESS), 2014 IEEE 19th International, 2014, pp. 1–4.

[31] Malešević NM, Maneski LZP, Ilić V, Jorgovanović N, Bijelić G, Keller T (2012). A multi-pad electrode based functional electrical stimulation system for restoration of grasp. J Neuro Eng Rehab.

[32] Maneski LZP, Malešević NM, Savić AM, Keller T, Popović DB (2013). Surface-distributed low-frequency asynchronous stimulation delays fatigue of stimulated muscles. Muscle Nerve.

[33] J. Axelgaard and S. Heard, “Medical electrode,” ed: Google Patents, 2000.

[34] K. Tuck, “Tilt sensing using linear accelerometers,” Freescale Semiconductor Application Note AN3107*,* 2007.

[35] Kennedy JC, Alexander IJ, Hayes KC (1982). Nerve supply of the human knee and its functional importance. Am J Sports Med.

